# Internal Herniation of the Transverse Colon and Stricture of the Gastric Body: A Case Report

**DOI:** 10.7759/cureus.56712

**Published:** 2024-03-22

**Authors:** Jacob B Morgan, Grace Corrier, Trevor Perry, Nicholas Hudspeth, Michael Garvie, Phillip Bradbury, Savannah Newell, John Lipka

**Affiliations:** 1 Medicine, Edward Via College of Osteopathic Medicine, Monroe, USA; 2 Anatomical Sciences, Edward Via College of Osteopathic Medicine, Monroe, USA; 3 Surgery, Edward Via College of Osteopathic Medicine, Monroe, USA

**Keywords:** anatomy, cadaveric findings, post-dissection findings, hernia of transverse colon, gastric stricture

## Abstract

Internal herniation is a protrusion of the bowel limited to the abdominal cavity. This pathology is rare and difficult to diagnose due to a wide array of symptoms that may manifest. Internal hernias have the potential to affect surrounding organs such as the stomach and adjacent bowel due to the compressive force of the protruding bowel. The effects of internal herniation commonly present in one of two ways: acute obstruction which requires emergent intervention and subacute, vague symptoms that are difficult to diagnose. This case presents the findings of a post-mortem dissection of a 92-year-old willed body donor. Dissection of the abdominal cavity revealed a large internal hernia of the transverse colon that communicated superiorly posterior to the stomach. As a result of the hernia, the stomach in this patient had a stricture of the gastric body. We assert that this stricture was formed over an extended period of time due to the lack of diagnosis and treatment of the internal hernia.

## Introduction

Bowel herniation has an incidence rate of 4-5% globally. Internal hernias are rare findings making up approximately 1% of all bowel hernias [1]. This form of herniation communicates through a mesenteric or peritoneal opening rather than through the external abdominal wall as seen in external herniation [2]. Internal hernias can present with symptoms ranging from subacute nonspecific symptoms to acute colicky obstruction with loss of flatus [3,4]. With a wide array of presentations and low incidence, this pathology can be challenging to diagnose with imaging [5].

The second finding presented in this case report is a stricture of the gastric body which is categorized under a broader diagnosis known as gastric outlet obstruction (GOO) [6]. GOO is classified with two etiologies: mechanical obstructions and motility disorders [7]. Mechanical obstruction, like that which is presented here, results from a physical blockage with the most common causes including peptic ulcer disease, polyps, anastomotic strictures, pancreatitis, and annular pancreas [3]. Rarer causes include internal herniation of bowel which was previously documented in a 69-year-old female suffering from GOO. On imaging, the causative agent was a portion of the cecal bowel which communicated through the foramen of Winslow and compressed the stomach [5].

## Case presentation

This case discusses the abdominal findings of a 92-year-old female following post-mortem dissection. The patient’s known medical history included failure to thrive (FTT), cerebral atherosclerosis, dementia, chronic obstructive pulmonary disorder, and breast cancer. Surgical history included prior cholecystectomy, appendectomy, and total hysterectomy. Of note, past medical history did not include diagnoses regarding the internal hernia or gastric stricture. On gross examination, the patient’s habitus was frail with appreciable muscle wasting and atrophy. General inspection of the abdomen revealed no distention, protrusions, or palpable masses. Additionally, there were no incisions or scarring on the stomach or overlying skin indicating previous bariatric surgery.

**Figure 1:**
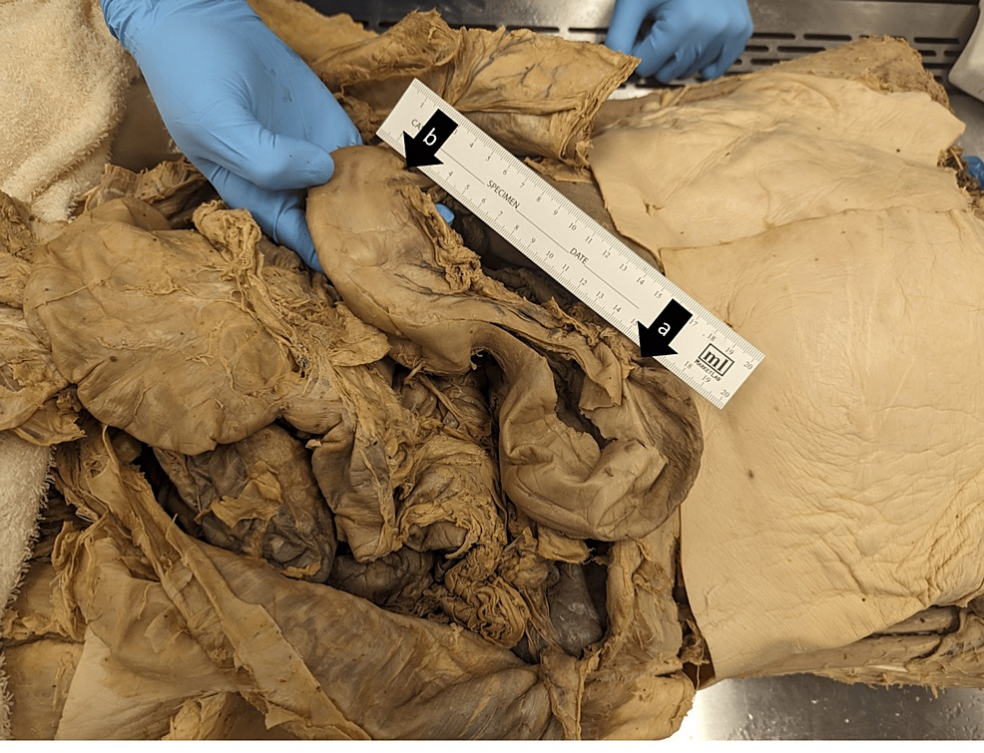
Gross imaging of the stomach Gross imaging of the stomach shows a stricture of the gastric body which creates a "peanut-shaped" appearance. Label A represents the esophagus. Label B represents the pylorus. The stricture’s lumen (not seen) was less than a centimeter on dissection. There was no discoloration or necrosis within the stricture.

The stomach presented with a stricture circumscribing the body giving it a "peanut-shaped" appearance (Figure 1). This stricture was approximately 5 cm proximal to the pyloric sphincter. The stricture measured 30 x 27 mm in contrast to the gastric body adjacent to the stricture which measured 90 mm at its widest point. The internal lumen of the stricture was less than a centimeter in diameter. There was no gross scarring, discoloration, or necrosis appreciated on the internal surface of the stricture. The rugae within the stricture were pronounced and consistent with the surrounding rugae.

**Figure 2:**
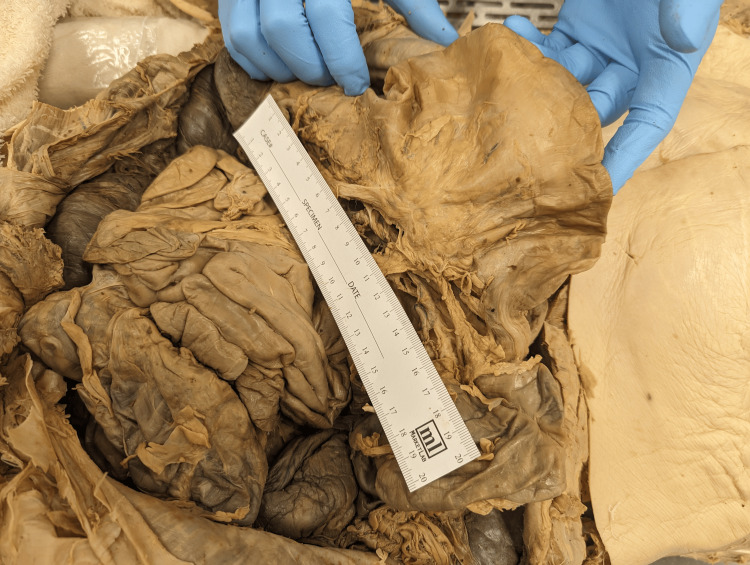
Gross imaging of the colonic hernia On gross examination, there was a large herniation of the transverse colon. The hernia measured 52 x 106 mm. The wall of the herniated colon was thin with no tears or breaks or discoloration. No necrosis was identified on examination.

Within the lesser sac, a space posterior to the lesser omentum, there was a thin-walled herniation stemming from the central third of the transverse colon (Figure 2). The hernia extended superiorly into the lesser sac and interfaced with the body of the stomach. It measured 52 x 106 mm. There was no discoloration or necrosis of the herniated colon. The surrounding greater and lesser omenta were intact prior to dissection with no signs of trauma or external protrusions from the hernia.

## Discussion

Internal hernias are labeled based on the location of the protruding bowel. The hernia identified in this patient extended through the foramen of Winslow into the lesser sac which is known as a foramen of Winslow hernia (FOWH). In typical anatomy, the lesser sac has a single opening connecting it to the greater peritoneal cavity known as the foramen of Winslow [8,9]. FOWH is rare, accounting for only 0.08% of all hernias and less than 200 cases have been documented in the literature since first described in 1834 [10,11]. The small bowel is responsible for approximately 65% of these herniations, while the transverse colon has been implicated in approximately 7% of known cases [10]. Anatomic abnormalities such as an enlarged foramen of Winslow and defects in the gastro-hepatic ligament can predispose patients to FOWH [6]. Furthermore, intra-abdominal pressure change and post-operative fibrosis that accompany intra-abdominal surgeries, including cholecystectomy, have also been identified as risk factors [5,11,12].

Internal hernias typically present in two ways: acute obstruction with possible strangulation or sub-acutely with vague, chronic symptoms [2,4]. Additionally, secondary organ involvement may present due to external compression from the herniated bowel. Abdominal pain, nausea, and vomiting are the most common documented symptoms; however, presentation varies in severity depending on intestinal and visceral involvement [1,8,11,12]. Multiple cases of internal herniation have been described in the literature with varying presentation. Crispin-Trebejo et al. record the case of a 63-year-old woman who presented with a long history of untreated chronic constipation [3]. The patient tested positive for abdominal distension and an eight-kilogram weight loss over the span of one month before hospitalization. History was positive for a bilateral oophorectomy 25 years prior to diagnosis. During hospitalization, an abdominal radiograph revealed colonic distension and air-fluid levels. The patient underwent an exploratory laparoscopy which revealed a trans-mesenteric internal hernia of the transverse colon.

In Hayes and Newton, a 69-year-old female presented with a one-day history of nausea, retching, and radiating left upper quadrant pain. Computed tomography revealed GOO which was initially thought to be secondary to a gastric duplication cyst. The patient underwent an exploratory laparotomy which revealed a portion of small bowel herniating through the foramen of Winslow. The presence of the bowel in the lesser sac was the causative agent of the patient's GOO symptoms. While both cases report internal herniation, the patients’ initial presentations are unique. In Crispin-Trebejo et al., the patient reported mild, chronic symptoms [3]. This is in contrast to the patient in Hayes and Newton who presented acutely with retching and abdominal pain.

Given the age of the presently discussed patient and the lack of documented abdominal complaints at the time of her death, it is presumed that her hernia was of a subacute nature. The intermittent character would not have impeded peristalsis entirely, allowing her to continue to pass flatus and stool as evidenced by the stool present in the distal colon upon dissection [10]. The significant stricture in the middle of her stomach can also be explained by the long-term extrinsic compression on the lesser curvature of the stomach from the herniated colon akin to the findings presented by Hayes and Newton [5].

Furthermore, it is possible that this gastric stricture was a contributing factor in her reduced body mass with the knowledge that she suffered from FTT and malnutrition at the time of death. This aligns with the findings in Crispin-Trebejo et al. which reported a significant weight loss secondary to the hernia [3]. The extrinsic compression caused by the hernia would have impaired normal gastric emptying and lead to early satiety, malnutrition, and weight loss [7]. The patient's diagnosis of dementia may have further obfuscated the clinical picture due to a diminished ability to convey the extent of symptoms, leading to misdiagnosis and progression of the hernia and stricture [13].

## Conclusions

The internal hernia was likely of a subacute nature based on the age of death and lack of documented abdominal complaints. The lack of detection and treatment of the hernia allowed it to precipitate a gastric stricture due to prolonged external compressive forces on the gastric body. The lack of premortem diagnosis of GOO and internal herniation may be partly attributed to the patient’s diagnoses of FTT and dementia which could have obscured the clinical picture. In summary, the combination of these findings coincides with previously documented cases and further illuminates the potential long-term, secondary effects of internal hernias. These secondary effects include gastric involvement and subsequent stricture if not detected and treated.
